# Factors leading to failure to diagnose pulmonary malignant tumors using endobronchial ultrasound with guide sheath within the target lesion

**DOI:** 10.1186/s12931-019-1178-8

**Published:** 2019-09-12

**Authors:** Yoichi Nishii, Taro Yasuma, Kentaro Ito, Yuta Suzuki, Fumiaki Watanabe, Tetsu Kobayashi, Kota Nishihama, Corina N. D’Alessandro-Gabazza, Hajime Fujimoto, Esteban C. Gabazza, Fumihiro Asano, Osamu Taguchi, Osamu Hataji

**Affiliations:** 1 Respiratory Center, Matsusaka Municipal Hospital, Tonomachi 1550, Matsusaka, Mie 515-8544 Japan; 2 Department of Immunology, Mie University Faculty and Graduate School of Medicine, Edobashi 2-174, Tsu-city, Mie 514-8507 Japan; 3 Department of Pulmonary and Critical Care Medicine, Mie University Faculty and Graduate School of Medicine, Edobashi 2-174, Tsu, Mie 514-8507 Japan; 4 Gifu Prefectural General Medical Center, Noisshiki 4-6-1, Gifu, Gifu 500-8717 Japan

**Keywords:** Bronchoscopy, Diagnostic errors, Lung, Ultrasonography, Neoplasms

## Abstract

**Background:**

The diagnostic yield of peripheral pulmonary lesions has significantly increased with the use of radial endobronchial ultrasound with guide sheath within the lesion. Here, we retrospectively evaluated factors leading to misdiagnosis of pulmonary malignant tumors using endobronchial ultrasound with the guide sheath within the lesion.

**Methods:**

We assessed the final histopathological diagnosis of biopsy samples taken from 130 patients with lung malignant tumors that underwent endobronchial ultrasound with guide sheath within the lesion.

**Results:**

Among 130 patients, 8 (6%) showed no definite malignant findings in biopsy samples but the presence of malignant cells (primary lung cancer 7, diffuse large B cell lymphoma 1) was subsequently confirmed by histopathological study of specimens taken by computed tomography-guided needle biopsy or surgery. Of the eight cases with diagnostic failure, the size of the biopsy sample was insufficient in five due to technical difficulties during the diagnostic procedure, and the diagnosis of malignant tumor was difficult in five cases because of extensive scarring tissue or central necrosis.

**Conclusions:**

The results of this study showed that technical difficulties and/or pathological heterogeneity of the tumor might lead to failure to diagnose lung malignant tumor in cases using endobronchial ultrasound with guide sheath within the lesion.

## Background

The procedure of bronchoscopy for tissue sampling of lung peripheral masses has been traditionally carried out under fluoroscopic guidance, and the bronchial branch connected to the lesion has been classically estimated from the 2-dimensional lung computed tomography images taken prior to the bronchoscopic study [1]. However, the diagnostic yield of peripheral pulmonary lesions of less than 2 cm is very low (34%) when this conventional bronchoscopic method is used [1–3]. Radial endobronchial ultrasound (EBUS) and virtual bronchoscopic navigation system have been recently developed to improve the diagnostic yield of bronchoscopic techniques [4]. Indeed, recent studies have shown that the combined use of both techniques have dramatically increased (> 80%) the diagnostic yield of peripheral pulmonary lesions of less than 2 cm [2, 3]. Further, the diagnostic yield is even higher when the EBUS shows that the guide sheath is within the target lesion [5, 6]. Nonetheless, about 10% of pulmonary malignant lesions are not diagnosed despite being the guide sheath within the lesion [4]. The cause of this diagnostic failure is unclear. The aim of the present study was to describe retrospectively the clinical and histopathological characteristics of cases with diagnostic failure after EBUS with the guide sheath within the lesion.

## Patients and methods

**Fig. 1:**
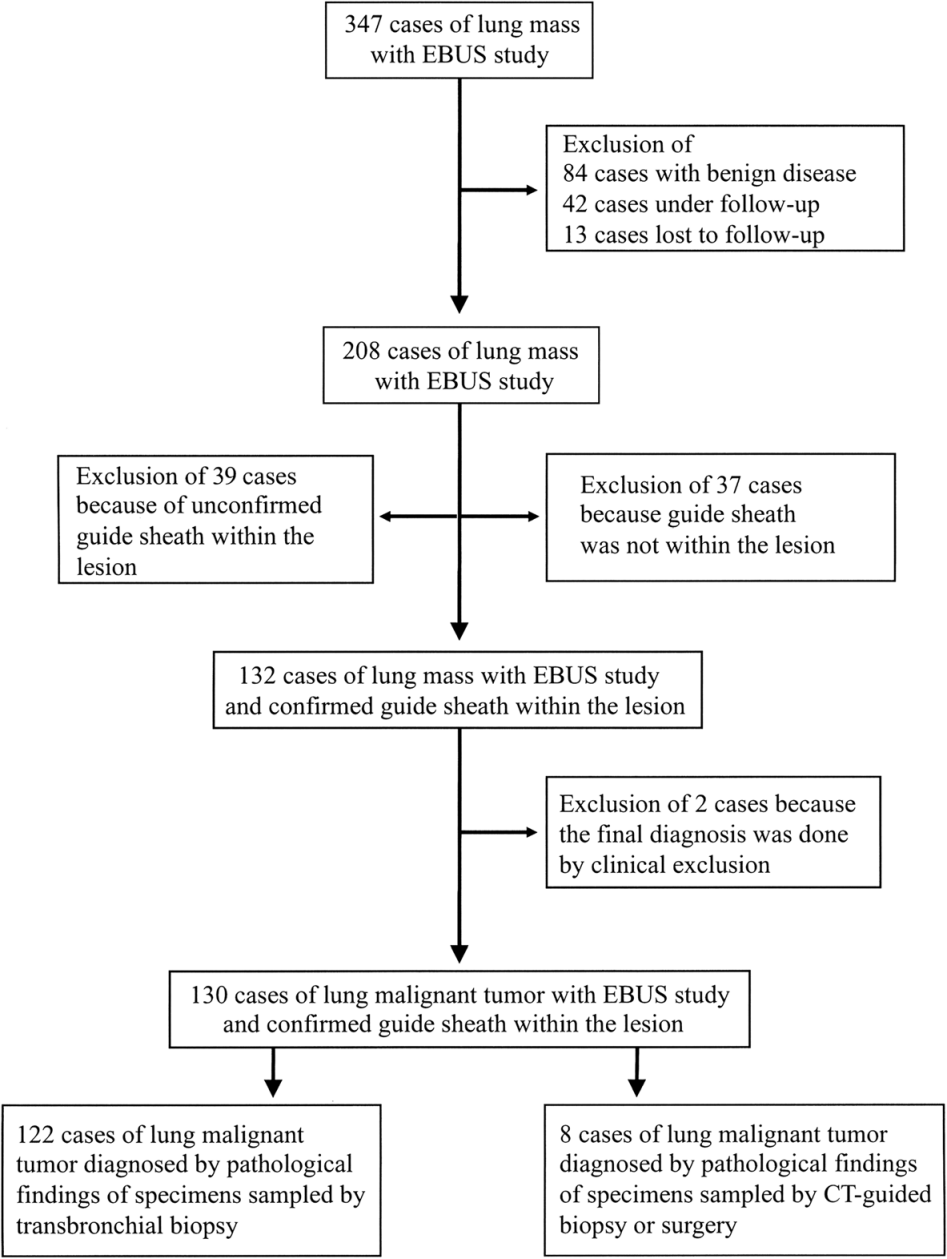
Study selection procedure. The records of 347 patients with lung mass that underwent endobronchial ultrasound with guide sheath were evaluated and 130 cases with confirmed diagnosis and 8 cases with misdiagnosis were selected following the described criteria

We retrospectively evaluated 347 patients with lung mass that underwent EBUS with guide sheath (EBUS-GS) at the Respiratory Center of Matsusaka Municipal Hospital between February 2015 and August 2016. Among 347 patients, 84 with benign lesion, 42 under follow-up, and 13 lost to follow-up were excluded from the study. Of the remaining 208 patients with lung mass, 26 with ground glass opacity and 13 with cavitation were excluded because it was difficult to confirm whether the guide sheath was within the lesion. During the EBUS-GS procedure performed in the remaining 169 patients, the guide sheath was within the target lesion in 132 cases, and among these, the histopathological study revealed malignant tumor in 122 cases and no malignant findings in 10 cases. Of these 10 cases, 2 were excluded because the final diagnosis of malignant tumor was possible only by clinical exclusion, and 8 underwent CT-guided needle biopsy or surgery for final diagnosis (Fig. 1). The histopathological diagnosis was malignant tumor in the 8 cases.

The bronchoscopic procedure was performed as previously described [2]. A flexible bronchoscope was inserted after spraying 2% lidocaine hydrochloride on the pharyngeal mucosa and intravenous administration of 2 mg midazolam (SANDOZ Japan, Tokyo). Additional injection of midazolam was performed as required. The flexible bronchoscope used in the study was Olympus BF Type P260F (Olympus Medical Systems, Tokyo, Japan) with an external diameter of 4 mm and a channel diameter of 2 mm or Olympus BF Type P290 (Olympus Medical Systems, Tokyo, Japan) with an external diameter of 4.2 mm and a channel diameter of 2 mm. Insertion of the bronchoscope was assisted using a virtual bronchoscopic navigation system (Bf-NAVI; Cybernet Systems, Tokyo) as described [7] . The virtual bronchoscopic images were prepared using the computed tomography scan (TSX-101A/QA, Canon Medical Corporation, Tochigi, Otawara, Japan) data of the lungs as previously described [2]. A radial type EBUS probe of 1.4 mm (UM-S20-17S; Olympus Medical Systems, Tokyo) was introduced with a guide sheath (SG-200C; Olympus Medical Systems, Tokyo, Japan) through an endoscopic channel to visualize the lung lesions. Lung tissue samples were collected using forceps of 1.5 mm (FB-233D;Olympus Medical Systems, Tokyo, Japan) inserted into the guide sheath. After confirming visualization of the lesion, the EBUS probe was withdrawn. Brushing and washing were performed after completing the transbronchial biopsy.

### Statistical analysis

Data are expressed as the median and range. The different between two groups with continuous variable was evaluated by student test and the difference in distribution by Fisher’s exact test. Statistical analyses were done using the Graph Prism version 8.0.1 (Graphpad Software, San Diego, CA). A *p* < 0.05 was considered as statistical significance.

## Results

**Fig. 2:**
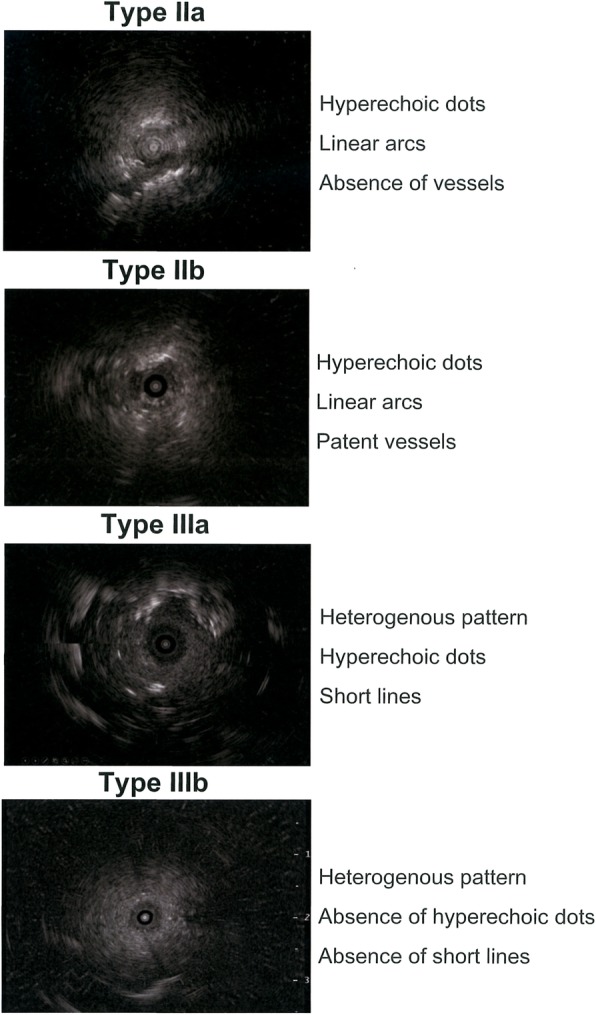
Endobronchial ultrasound patterns in patients with misdiagnosis. The findings of the radial endobronchial ultrasound in 8 patients with misdiagnosis were grouped according to the classification reported by Kurimoto et al

**Fig. 3:**
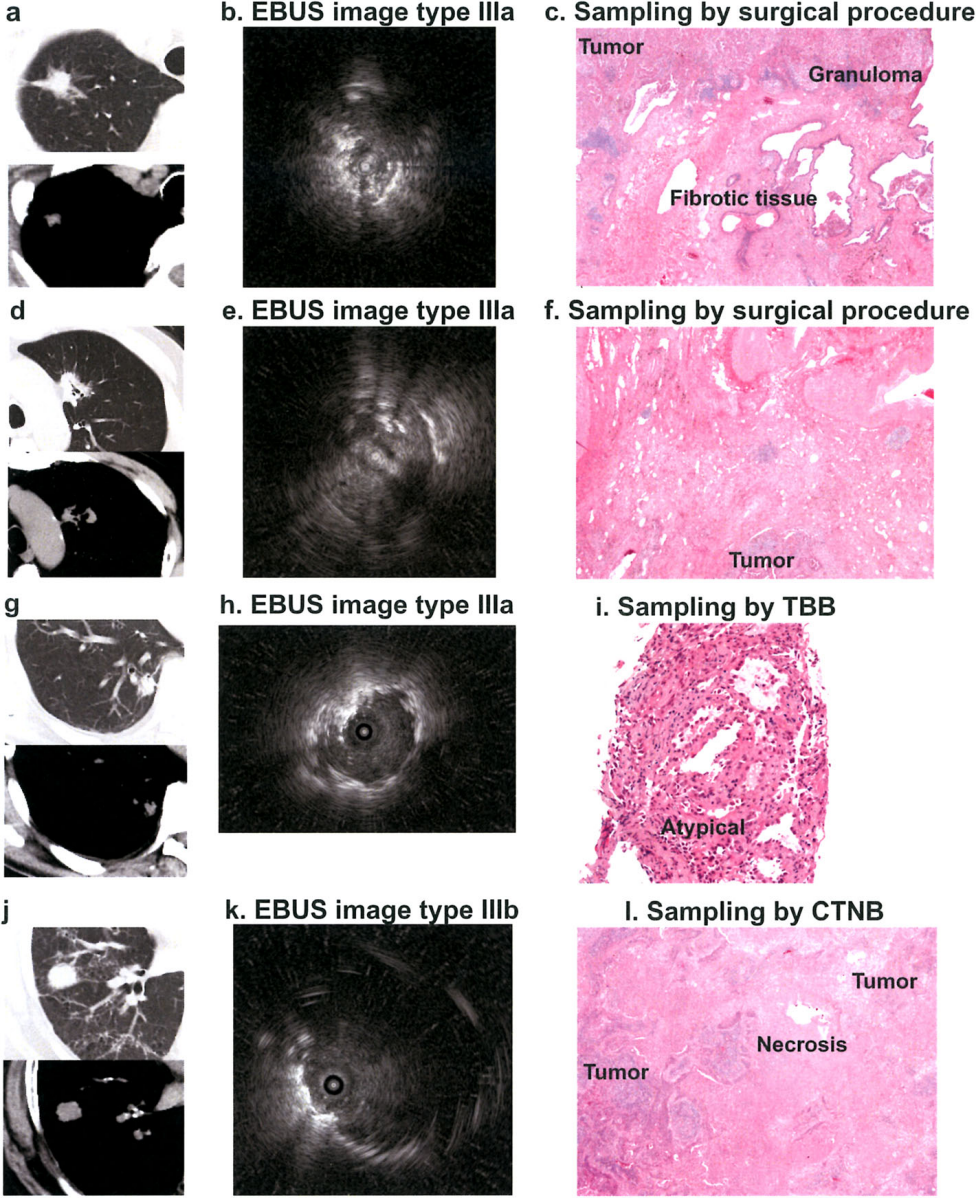
Computed tomography, endobronchial ultrasound and histopathological findings. Case 3 (**a**, **b**, **c**): tumor with fibrotic and granulomatous tissue. Case 4 (**d**, **e**, **f**): tumor with areas of fibrosis and necrosis. Case 5 (**g**, **h**, **i**): tissue sampling by transbronchial biopsy with tissue showing atypical cells. Case 6 (**j**, **k**, **l**): tumor with central necrosis. EBUS, endobronchial ultrasound; CTNB, CT-guided needle biopsy

The backgrounds and characteristics of the patients are described in Table 1. There was no significant different in the age, sex distribution, tumor size, tumor location or in the number of times the virtual bronchoscopic navigation and rapid on site cytology was performed between misdiagnosis and correct diagnosis groups (Table 1). The lesions were grouped according to EBUS findings based on the classification proposed by Kurimoto et al. (Fig. 2) as follows: type IIa (hyperechoic dots and linear arcs without vessels; *n* = 1), type IIb (hyperechoic dots and linear arcs with patent vessels; *n* = 1), type IIIa (heterogeneous pattern with hyperechoic dots and short lines; *n* = 4) and IIIb (heterogeneous pattern without hyperechoic dots or short lines; *n* = 2) (Fig. 3) [8]. EBUS images showed heterogeneous patterns in cases 3 to 8, and there was significant difference in the EBUS pattern between both misdiagnosis and correct diagnosis groups (Table 1). There were 7 cases of primary lung cancer and 1 case of diffuse large B cell lymphoma but there was no significant difference in the final diagnosis between both misdiagnosis and correct diagnosis groups (Table 1).

**Table 1:** Characteristics of the patients

Variables	Cases with Misdiagnosis (*n* = 8)	Cases with correct diagnosis (*n* = 122)	*P* values
Median age (range)	73 (37–83)	74 (31–95)	0.76
Sex			0.27
Male	4	84	
Female	4	38	
Lesion size (mm, median range)	27.8 (15.0–48.0)	32.9 (5.5–81.0)	0.30
Location			0.058
Central	0	11	
Intermediate	0	41	
Peripheral	8	70	
Virtual bronchoscopic navigation, n(%)	8 (100%)	101 (82.8%)	0.35
Rapid on site cytology, n(%)	7 (87.5%)	89 (73.0%)	0.62
EBUS-image			0.04
Type IIa	1	0	
Type IIb	1	4	
Type IIIa	4	57	
Type IIIb	2	57	
Unknown	0	4	
Final diagnosis			0.32
Primary lung cancer	7	117	
Malignant lymphoma	1	0	
Metastatic lung cancer	0	5

Transbronchial biopsy was conducted three times in one case and over five times in the other cases. The diagnostic report of cytological smear disclosed negative findings in cases 1, 2, 3 and 6, presence of atypical cells in cases 5 and 7, and suspicious findings of cancer in cases 4 and 8. The report of histopathological examination of biopsy samples collected during bronchoscopy and EBUS-GS showed negative findings in cases 2 and 3, atypical cells in cases 1, 3, 5 and 7, and insufficient amount of tissue in cases 2 and 8. During the bronchoscopic procedure, case 1 had persistent cough, cases 2 and 8 showed irregular respiratory rhythms and cases 1, 2, 4 and 5 presented bronchial bifurcations with steep angles. Tissue sampling was not technically difficult in cases 3, 6 and 7 (Table 2).

**Table 2:** Factors associated with diagnostic failure

Cases	Bronchi	EBUS-i	Diagnosis	Technical factors	Pathological factors
1	LtB1 + 2a	IIa	WDadeno	Bending and difficult stabilization of guide sheath	Tumor with central necrosis
2	RtB6c	IIb	MD adeno	Bending and difficult stabilization of guide sheath	Homogeneous distribution of tumor cells
3	RtB1bii	IIIa	MD adeno	No difficulty	Heterogeneous tissue. Tumor cells in granulomatous and fibrotic tissue
4	LtB3ci	IIIa	MD adeno	Bending of guide sheath	Tumor central necrosis
5	RtB10bii	IIIa	WD adeno	Bending and difficult stabilization of guide sheath	Tumor cells with predominant distribution in central areas
6	RtB4aiβ	IIIb	PD SCC	No difficulty	Tumor with central necrosis
7	RtB2bii	IIIa	WDadeno	No difficulty	Tumor with central necrosis
8	LtB8biiα	IIIb	DLBCL	Difficult stabilization of guide sheath	Homogeneous distribution of tumor cells

*EBUS-1* endobronchial ultrasound image, *LtB* left bronchus, *RtB* right bronchus, *adeno* adenocarcinoma, *SCC* squamous cell carcinoma, *WD* well-differentiated, *MD* moderately differentiated, *PD* poorly differentiated, *DLBCL* diffuse large B cell lymphoma

Histopathological examination of lung tissue specimens obtained by CT-guided needle biopsy or during surgery disclosed tumor central necrosis in cases 1, 4, 6 and 7, extensive areas of granulomatous and fibrotic tissue in case 3, lack of cancer cells in peripheral areas in case 5, and homogeneous distribution of cancer cells in cases 2 and 8 (Table 2**;** Fig. 3).

## Discussion

The diagnostic yield of peripheral pulmonary lesions of less than 3 cm by bronchoscopy has substantially improved with the use of R-EBUS or virtual bronchoscopic navigation reaching 60 to 80% when both virtual bronchoscopic navigation and EBUS-GS are used in combination [4, 9]. The diagnostic yield is particularly high when the EBUS guide sheath is placed within the target lesion [4]. However, misdiagnosis has been reported even in cases with the guide sheath within the lesion but the cause remains unclear. Here, we reported 8 cases of diagnostic failure despite being the guide sheath within the tumor site. Six patients (cases 3, 4, 5, 6, 7, 8) from the misdiagnosis group showed heterogeneous EBUS images and the EBUS pattern was significantly different between both misdiagnosis and correct diagnosis groups. Further, six patients (cases 1, 3, 4, 5, 6, 7) of the misdiagnosis group also showed inhomogeneous distribution of malignant cells within the tumor. Therefore, heterogeneity in the EBUS findings and in the intra-tumoral distribution of malignant cells appears to be important factors leading to failure of diagnosis. Sampling of large-sized or multiple specimens may improve the diagnostic yield. Yamada et al reported diagnostic yield of 96.5% in cases with 5 biopsied specimens and 100% in cases with 10 specimens [6]. The use of large forceps or cryobiopsy may also be an alternative approach to collect large-sized specimens [10, 11].

We found here that technical difficulty is another important cause of misdiagnosis. Sample collection was difficult in 5 patients (cases 1, 2, 4, 5) because steep angle of the bronchial branch restrained complete bending of the bronchoscope tip. In these cases, the use of ultra-thin bronchoscope in combination with R-BUS may help to overcome technical difficulties and to improve diagnostic yield as reported by Oki et al [12]. We also experienced technical difficulty in one patient with persistent cough (case 1) and in 2 patients with irregular respiratory rhythm (cases 2, 8). We believe that insufficient sedation during the bronchoscopic procedure could have been the cause. In this regards, the guidelines of the British Thoracic Society recommends sedation with benzodiazepines or/and opioids to improve tolerance and facilitate diagnostic procedures during bronchoscopy [13]. Adequate sedation can prevent throat reflex and coughing and facilitate specimen sampling [13].

## Conclusion

In brief, the results of this study showed that technical difficulties and/or heterogeneity of EBUS and histopathological findings of the tumor might lead to failure to diagnose lung malignant tumor even in cases using EBUS with the guide sheath within the tumor. The cause of diagnosis failure reported in this study may serve as important hints for developing countermeasures to improve the diagnostic yield in patients with lung peripheral malignant tumors.

## Data Availability

The data used and/or analyzed during the current study are available from the corresponding author on reasonable request.

## Abbreviations

adeno: Adenocarcinoma
CT: Computed tomography
DLBCL: Diffuse large B cell lymphoma
EBUS-i: Endobronchial ultrasound image
GS: Guide sheath
LtB: Left bronchus
MD: Moderately differentiated
PD: Poorly differentiated
R-EBUS: Radial endobronchial ultrasound
RtB: Right bronchus
SCC: Squamous cell carcinoma
WD: Well-differentiated
